# Lower MeDiC score is associated with non-referral to multidisciplinary team meeting discussion in bladder cancer patients: a nationwide and population-based study

**DOI:** 10.2340/1651-226X.2025.42756

**Published:** 2025-05-05

**Authors:** Jessica Wihl, Oskar Hagberg, Firas Aljabery, Truls Gårdmark, Abolfazl Hosseini, Staffan Jahnson, Tomas Jerlström, Viveka Ströck, Karin Söderkvist, Anders Ullén, Lars Holmberg, Christel Häggström, Fredrik Liedberg

**Affiliations:** a Department of Clinical Sciences, Division of Oncology and Pathology, Lund University, Lund, Sweden; b Regional Cancer Centre South, Region Skåne, Lund, Sweden; c Department of Hematology, Oncology and Radiation Physics, Skåne University Hospital, Lund, Sweden; d Department of Translational Medicine, Lund University, Malmö, Sweden; e Department of Clinical and Experimental Medicine, Division of Urology, Linköping University, Linköping, Sweden; f Department of Clinical Sciences, Danderyd Hospital, Karolinska Institute, Stockholm, Sweden; g Department of Molecular Medicine and Surgery, Karolinska Institutet, Stockholm, Sweden; h Department of Urology, School of Medical Sciences, Faculty of Medicine and Health, Örebro University, Örebro, Sweden; i Department of Urology, Sahlgrenska University Hospital and Institute of Clinical Sciences, Sahlgrenska Academy, University of Gothenburg, Sweden; j Department of Diagnostics and Intervention, Umeå University, Sweden; k Department of Oncology-Pathology, Karolinska Institutet, Stockholm, Sweden; l Department of Pelvic Cancer, Genitourinary Oncology and Urology Unit, Karolinska University Hospital, Stockholm, Sweden; m Department of Surgical Sciences, Uppsala University, Uppsala, Sweden; n School of Cancer and Pharmaceutical Sciences, King’s College London, London, UK; o Department of Diagnostics and Intervention, Registry Center North, Umeå University, Umeå, Sweden; p Department of Urology, Skåne University Hospital, Malmö, Sweden

**Keywords:** Bladder cancer, multidisciplinary team meeting, treatment recommendation, guidelines, scoring system, complexity factors

## Abstract

**Background and purpose:**

The Measure of Case-Discussion Complexity (MeDiC) tool was created to gauge case complexity at multidisciplinary team meetings (MDTM) for case selection and prioritization. We aimed to assess applicability and association with MeDiC score and non-compliance with national guideline-recommendations for MDTM referral in a bladder cancer setting.

**Material and methods:**

A modified MeDiC scoring system was applied in 8955 subjects with localized (T1-T4N0M0) or metastasized disease as per the Bladder Cancer Data Base Sweden (BladderBaSe) 2.0. Association between MeDiC score and not being discussed at MDTM was investigated by multivariable logistic regression, and further explored in relation to calendar time period, healthcare region, age at diagnosis and hospital volume.

**Results and interpretation:**

Median total MeDiC score was lower in individuals not being discussed at an MDTM (7.0 Inter Quartile Range [IQR] 6.0–9.0) compared to those who were (8.0 IQR 6.0–10.0). Adjusted odds ratio for not being discussed at an MDTM was 2.1 (95% confidence interval [CI] 1.8–2.4) for a MeDiC score in the lower quartile, as compared to the highest quartile, with higher estimates when performing stratified analyses in later calendar years and in specific healthcare regions. Our data indicate that the MeDiC score is applicable in bladder cancer patients, and we identified an association between lower MeDiC score and not being discussed at an MDTM.

## Introduction

The Measure of Case-Discussion Complexity (MeDiC) tool was developed to increase the understanding of different aspects of case complexity [1]. MeDiC may be used to ensure balanced prioritization strategies and as a clinical quality assurance for multidisciplinary team meeting (MDTM) management and treatment planning.

Among all urological malignancies, bladder cancer is the most frequently subjected to altered diagnosis and/or treatment after referral to MDTM [2–4]. Since May 2013, the Swedish National Guidelines on Urothelial Carcinoma recommend an MDTM discussion for all invasive bladder cancer patients with stage T1 and above, including those with metastasized disease [5]. Thus, patients with stage Ta and carcinoma in situ (approximately 50% of all bladder cancer patients) are treated according to guidelines but without need of MDTM discussion. Concerns about prolonged waiting times to MDTM and patient safety related to limited case discussion time slots have been raised in different types of cancers [6–14]. More vulnerable bladder cancer patients (advanced age, stage and/or poorer performance status) have been reported to less frequently being referred to MDTM [15]. Theoretically, applying the MeDiC score in bladder cancer could help improve patient prioritization.

To study the possible clinical applicability of the MeDiC score, we applied a modified version of the score to patients diagnosed with bladder cancer in the Bladder Cancer Data Base Sweden (BladderBaSe) 2.0 [16] and investigated if MeDiC score was associated with not being referred to MDTM discussion. Additionally, we performed stratified analyses to disentangle if associations were modified by age at diagnosis, calendar year, hospital volume or healthcare region.

## Material and methods

### Bladder Cancer Data Base Sweden 2.0, study population, and selected variables

Patients in the Swedish National Urinary Bladder Cancer Register (SNRUBC) have been linked to registers at the National Board of Health and Welfare and the Statistics Sweden to form BladderBaSe 2.0 [14]. To calculate a drug comorbidity index (DCI) [17] in BladderBaSe 2.0, we retrieved data from the Prescribed Drug Register. From Statistics Sweden we also retrieved data on the highest education level. In BladderBaSe 2.0, we ascertained data on age, gender, grade, clinical T stage, clinical N stage, M stage at diagnosis, and healthcare region from the SNRUBC. The study population consisted of all patients diagnosed with bladder cancer stage T1–T4 and/or lymph node metastases and/or distant metastases from August 1st, 2008, from when SNRUBC registered whether patients were discussed at an MDTM until December 31st, 2019. To quantify hospital volume while accounting for time trends, we assessed period-specific mean annual volume (PSMAV) dynamically by using data on all patients fulfilling the inclusion criteria specified earlier in the text. We defined PSMAV as the volume per year for the preceding 3 years, and a PSMAV was calculated separately for each hospital unit and year of diagnosis for the individual patient [18].

### The Measure of Case-Discussion Complexity score and outcome definitions

The MeDiC tool was developed by multidisciplinary team experts in the UK to assess cancer case complexity as a screening instrument for MDTM consideration through case selection and prioritization [1]. Originally developed for breast, colorectal, and gynecological cancer, the MeDiC score has also previously been evaluated in prostate cancer patients [19]. Individual patient MeDiC scores were compared to preformed clinical MDTM case-selection processes and key MDTM predictive factors were identified correlating with MeDiC scoring [19]. The MeDiC tool measures clinical and logistical complexities and consists of a template of 26 complexity items divided into three main categories: pathology, patient factors, and treatment factors. The items are each differently weighted, between 1 to 4 points for complexity following predefined descriptions [1]. We adapted the scoring system for the 26 complexity items in the MeDiC tool template to indicators of the variables in BladderBaSe 2.0 [16, 17]. Following the predefined description of the MeDiC tool [1], items were weighted between 1 to 4 points for complexity. The exact definition and weight for each item are presented in Supplementary Table 1. Information on items 10, 12, and 17–24 was not available in BladderBaSe 2.0., and therefore missing in the study population, although item 22 (unusual anatomy) is not applicable in a bladder cancer context. Three of the original MeDiC 26 items (item no. 3, 4, and 5) are not relevant in the bladder cancer context, and consequently all patients scored 0 in these items. Our modified version of the MeDiC score was calculated for all patients with a minimum score of 3, as a consequence of that all patients fulfilled one point for item 1, 6, and 26, whereas theoretically the maximum score was 28. The primary outcome measure was to not being discussed at an MDTM.

### Statistical methods

For each individual in the study population a modified MeDiC score was calculated. For the total study population, and in quartiles of the MeDiC score, categorical variables were presented as proportions, and age was presented with medians and interquartile ranges (IQR). To visualize the distribution of patients not being discussed at an MDTM, we displayed the proportion of not being discussed at an MDTM over calendar time.

Associations between MeDiC score and not being discussed at an MDTM were investigated in a multivariable logistic regression model adjusting for age and gender with the MeDiC score in quartiles, and the highest quartile used as reference by calculating odds ratios (ORs) with 95% confidence intervals (CIs). Given that age and gender parameters are not included in the MeDiC score and motivate these factors as adjustment factors, we checked proportions of not being discussed at an MDT by age in tertiles and gender.

To explore interaction, we applied a similar logistic regression model in subgroups by calendar year of diagnosis (2008–2013 and 2014–2019 (as MDTM-discussion was recommended for all patients with stage T1-T4 and those with metastasized disease from 2014 and onwards)), healthcare region, age at diagnosis in tertiles (<68/68–75/≥76), and PSMAV (in tertiles), respectively. Analyses were adjusted for age and gender, except for the subgroup analysis by age, where adjustment was performed for gender only. A formal likelihood ratio test was also performed to assess differences between the regression models. Additionally, we used the same logistic regression model to assess the potential linear association between the MeDiC score and the probability of not being discussed at an MDTM, calculating ORs for each one-unit decrease in the MeDiC score. The R statistical package version 4.3.0 was used for all analyses.

### Ethical aspects

This study was approved by The Research Ethics Board at Uppsala University, Sweden (Dnr 2015-277, 2019-03574, 2020-05123, and 2022-01747-02).

## Results

**Figure 1:**
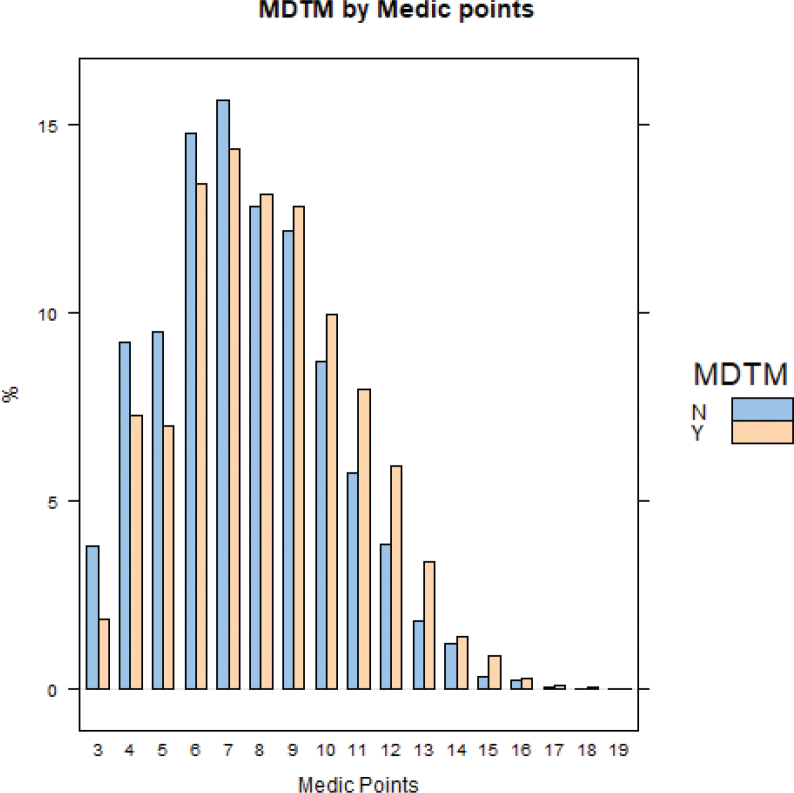
Distribution of MeDiC-points in patients not discussed at MDTM (N) and those who were discussed (Y), respectively.

**Figure 2:**
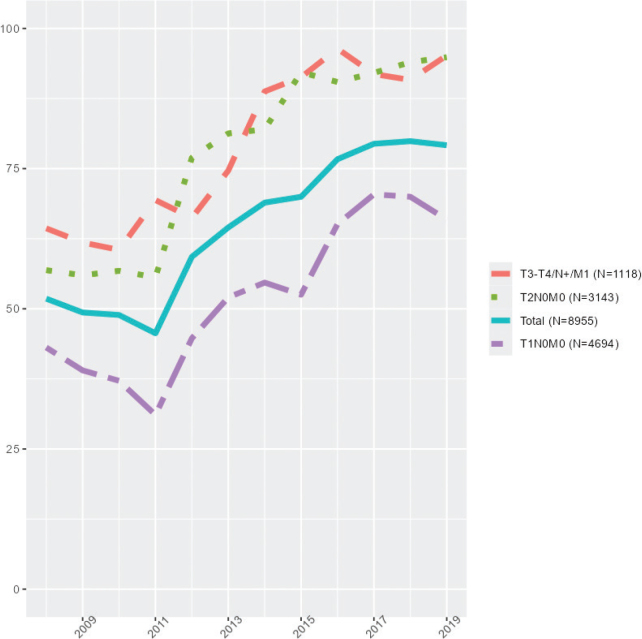
Registration of proportions (%) discussed at an MDTM over time from August 1st 2008 to December 31st 2019.

Our study included 8955 patients with distribution of MeDiC score by referral to MDTM discussion (Figure 1). The median MeDiC score was lower in individuals not discussed at an MDTM 7.0 (IQR 6.0–9.0) versus 8.0 (IQR 6.0–10.0). From August 2008 to 2019 the proportion of patients with tumor stages T1–T4 or metastasized disease not being discussed at an MDTM declined sharply in 2013 (Figure 2). The median age of the studied population was 72 (IQR 66–78) years and 24% were females. Further data of patient characteristics, pathologic and treatment factors separated by MeDiC score in quartiles are given in Table 1. Of patients in the highest MeDiC score quartile, 26% were not discussed at an MDTM whereas 40% were not discussed in the lowest quartile. A larger proportion of patients ≥76 years was not discussed at MDTM, as compared to younger ages (Supplementary Table 2).

**Table 1:** Distribution of patient, pathologic and treatment factors between MeDiC scores in quartiles.

MeDiC score (quartiles)	3–6	7–8	9–10	11–19	Total
Numbers (Row %)	2884 (32)	2493 (28)	1980 (22)	1598 (18)	8955
**MDTM**					
No	1141 (37)	871 (28)	638 (21)	408 (13)	3058
Yes	1743 (30)	1622 (28)	1342 (23)	1190 (20)	5897
Median age (IQR) years	70 (63–75)	72 (66–78)	73 (67–79)	76 (70–81)	72 (66–78)
**Sex**					
Female	669 (31)	567 (27)	476 (22)	426 (20)	2138
Male	2215 (32)	1926 (28)	1504 (22)	1172 (17)	6817
**Grade WHO 1999**					
G1/LMP	38 (60)	14 (22)	6 (10)	5 (8)	63
G2	929 (52)	447 (25)	267 (15)	142 (8)	1785
G3-G4/anaplastic	1825 (27)	1922 (28)	1634 (24)	1379 (20)	6760
Gx	92	110	73	72	347
**Clinical T-stage group**					
T1	2329 (50)	1321 (28)	758 (16)	286 (6)	4694
T2	460 (15)	877 (28)	886 (28)	920 (29)	3143
T3-T4 and/or N+ and/or M1	95 (8)	295 (26)	336 (30)	392 (35)	1118
**Clinical N-stage**					
N0/Nx	2865 (34)	2358 (28)	1837 (22)	1398 (17)	8458
N1	5 (2)	49 (24)	59 (29)	88 (44)	201
N2	10 (5)	55 (28)	57 (29)	76 (38)	198
N3	2 (2)	31 (33)	26 (28)	35 (37)	94
**Clinical M-stage**					
M0/MX	2863 (33)	2403 (28)	1872 (22)	1457 (17)	8595
M1	19 (5)	90 (25)	107 (30)	141 (39)	357
**Previous cancer within 10 years of bladder cancer diagnosis**					
N	2727 (35)	2218 (28)	1691 (22)	1153 (15)	7789
Y	157 (13)	275 (24)	289 (25)	445 (38)	1166
**Previous abdominal surgery**					
N	2880 (34)	2439 (29)	1850 (22)	1227 (15)	8396
Y	4 (1)	54 (10)	130 (23)	371 (66)	559
**DCI (tertiles)**					
−0.75	1878 (53)	948 (27)	521 (15)	172 (5)	3519
0.75–2	784 (28)	892 (32)	582 (21)	514 (19)	2772
2-	222 (8)	653 (25)	877 (33)	912 (34)	2664
**Mental comorbidities**					
N	2759 (36)	2145 (28)	1657 (21)	1156 (15)	7717
Y	125 (10)	348 (28)	323 (26)	442 (36)	1238
Educational level*					
Low	116 (4)	715 (22)	1165 (35)	1289 (39)	3285
Middle	1761 (48)	1160 (31)	572 (15)	200 (5)	3693
High	955 (51)	586 (31)	227 (12)	104 (6)	1872
Increased risk of treatment toxicity**					
N	2884 (33)	2479 (29)	1912 (22)	1355 (16)	8630
Y	0 (0)	14 (4)	68 (21)	243 (75)	325
**PSMAV (tertiles)**					
-49	938 (31)	824 (27)	695 (23)	552 (18)	3009
49–77	1001 (34)	783 (26)	631 (21)	540 (18)	2955
77–229	945 (32)	886 (30)	654 (22)	506 (17)	2991
**Healthcare region**					
Stockholm	573 (35)	468 (29)	327 (20)	253 (16)	1621
Mid Sweden	651 (33)	513 (26)	453 (23)	357 (18)	1974
Southeastern	339 (31)	308 (28)	248 (22)	216 (19)	1111
Southern	595 (31)	547 (29)	436 (23)	326 (17)	1904
Western	474 (31)	445 (29)	325 (21)	281 (18)	1525
Northern	252 (31)	212 (26)	191 (23)	165 (20)	820

MDTM: multidisciplinary team meeting; IQR: Inter Quartile Range; LMP: Low malignant potential; NA: Not available; DCI: Drug Comorbidity Index; PSMAV: Period Specific Mean Annual Volume in hospitals where the individual patient was diagnosed with bladder cancer.

Grey rows represent items included when calculating MeDiC-points.

* Missing data for 105 individuals.

** Cystectomy in an individual 80 years and above or neoadjuvant chemotherapy in an individual 76 years and above.

In a logistic regression model adjusting for age and gender, a MeDiC score in the lowest quartile, as compared to highest, was associated with not being discussed at an MDTM, OR of 2.1 (95% CI 1.8–2.4) (Table 2).

**Table 2:** Odds ratios for not being discussed at a multidisciplinary team meeting (MDTM) derived from a logistic regression model adjusting for age and gender.

MeDiC points	Patients	Patients not discussed at MDTM (%)	Odds ratio (95% CI)
11–19	1598	408 (26)	1 (reference)
9–10	1980	638 (32)	1.4 (1.2–1.7)
7–8	2493	871 (35)	1.7 (1.4–1.9)
3–6	2884	1141 (40)	2.1 (1.8–2.4)

CI: confidence interval.

The association between MeDiC score and not being discussed at an MDTM was modified by calendar year of diagnosis (*p* = 0.004), with larger estimates during the later years for lower MeDiC scores (Supplementary Table 3). Furthermore, the healthcare region interacted with the association between MeDiC score and not being discussed at an MDTM (*p* < 0.001). We found larger estimates for not being discussed at an MDTM in lower MeDiC scores in the Southeastern and Northern healthcare regions (data not shown). No interactions were identified between MeDiC score and not being discussed at MDTM for age at diagnosis and PSMAV.

## Discussion

In this nationwide population-based study utilizing BladderBase 2.0, a lower MeDiC score was associated with not being discussed at an MDTM. The association was reinforced during the second half of the study period (2014–2019) and the estimates were higher in the Southeastern and Northern healthcare regions when corresponding stratified analyses were performed.

To the best of our knowledge this is the first study using a modified version of the MeDiC tool in the context of bladder cancer, and also the first study that includes a large number of patients (*n* = 8,955) from an unselected population. The study is limited by lacking some of the original MeDiC items due to retrospective setting and lack of information in the dataset (Supplementary Table 1). In a future prospective setting, these items need to be included to ensure granular assessment of bladder cancer patient complexity in the clinical situation, to adequately validate the MeDiC score in bladder cancer patients.

Although current Swedish guidelines on urothelial carcinoma [5] recommend MDTM for all the investigated patients, this study suggests that urologists and oncologists refrain from referring less complex bladder cancer patients to MDTM, and especially so from 2014 and onwards. Whether this is related to MDTM capacity issues during later years or varying MDTM-capacity between healthcare regions in Sweden or practice patterns, is unclear. The MeDiC score has hitherto not been applied in Swedish bladder cancer care. However, the association between lower MeDiC scores with lower probability of MDTM referral in our retrospective investigation indicate that the MeDiC scoring system reflects a practice to prioritize more complex patients for MDTM referral. Refraining from MDTM referral for stage Ta is already recommended by Swedish national guidelines [5]. Still, not referring 26% of patients with a MeDiC score in the highest quartile among those recommended by guidelines for MDTM referral is a too high figure, and these patients may miss out on being offered a treatment tailored to their needs. Whether a prospective application of MeDiC score can decrease the proportion of those 26% not referred to MDTM, despite a complex situation, needs to be further studied.

According to a former study physicians cannot reliably predict which patients that will have an altered treatment plan after a urooncologic MDTM [20]. The MeDiC score could be one supplementary method to prioritize bladder cancer patients for MDTM, especially when resources are limited. However, while the MeDiC score considers tumor, treatment, and patient factors, it does not – importantly in bladder cancer – include age [1]. In a recent Dutch study higher age was associated with not being discussed at an MDTM [15]. In that population-based study, not being referred to MDTM was also associated with a lower probability of receiving curative treatment [15]. However, older patients might especially benefit from MDTM referral. They could be offered curative bladder-sparing treatments, be eligible for new combinations of complex oncologic systemic treatment options [21, 22], and for other palliative treatment modalities with local radiation [23] or even urinary diversion. In our study, age was not used to discriminate between patients, that is did not modify the association between MeDiC score and not being discussed at an MDTM. We were unable to define older patients not referred for MDTM suitable for the above-mentioned treatment options in our study. Further studies are needed to disentangle if and how age needs to be considered if MeDiC score is applied to prioritize bladder cancer patients for MDTM-referral.

Another possible drawback by using MeDiC scores to prioritize referral to MDTM is that referral of patients with less advanced stage T1-disease, where a pathological stage review at MDTM has been reported to frequently (20%) both up- and downstage the primary pathology and such altered tumor stage after pathology review was associated with progression-free survival [24]. Thus, as stage T1-patients receive a lower MeDiC score compared to patients with more advanced tumors, one cannot rule out that prioritizing individual patients to MDTM referral strictly by MeDiC score might result in both missed radical treatment opportunities or overtreatment for some patients.

## Conclusion

The association between a low MeDiC score and not being referred to an MDTM seems to reflect a rational policy to prioritize more complex patients to MDTM, but our data still indicate that many patients might miss out on being offered personalized treatment through a MDTM-referral. Our findings also indicate that the MeDiC score may be a help in MDTM prioritization for bladder cancer patients in the future. For further understanding of its role and how age should be integrated in a score for bladder cancer, prospective studies defining optimal cut-off scores to avoid undertreatment are needed.

## Author contributions

Conceptualization, methodology, formal analysis, and validation: JW, OH, CH, FL. Investigation: JW, OH, FL. Resources: FL. Data curation and visualization: OH, FL. Writing, original draft preparation: JW, FL. Writing, review, and editing: all authors.

All authors have read and agreed to the published version of the manuscript.

## Data availability statement

Data are available on reasonable request. Reports from the SNRUBC are available online (only in Swedish). Researchers can apply for data by submitting a proposal to the BladderBaSe 2.0 steering committee and data files for studies can be uploaded to remote servers for secure analysis. For more information contact the corresponding author.

## Supplementary Material

Lower MeDiC score is associated with non-referral to multidisciplinary team meeting discussion in bladder cancer patients: a nationwide and population-based study

Lower MeDiC score is associated with non-referral to multidisciplinary team meeting discussion in bladder cancer patients: a nationwide and population-based study
