# Supplementary material for: Lower MeDiC score is associated with non-referral to multidisciplinary team meeting discussion in bladder cancer patients: a nationwide and population-based study
Source: Acta Oncol. 2025 May 5;64:42756. doi: 10.2340/1651-226X.2025.42756 (PMC12067983; doi:10.2340/1651-226X.2025.42756)
Supplement: Lower MeDiC score is associated with non-referral to multidisciplinary team meeting discussion in bladder cancer patients: a nationwide and population-based study [file AO-64-42756-s2.pdf]

Supplementary material has been published as submitted. It has not been copyedited, or typeset by Acta Oncologica

		Numbers	Numbers no MDTM (%)	Number MDTM (%)
Age in tertiles (years)	<68	2730	883 (32%)	1847 (68%)
	68-75	3064	992 (32%)	2072 (68%)
	>=76	3161	1183 (37%)	1978 (63%)
Gender	Male	6817	2348 (34%)	4469 (66%)
	Female	2138	710 (33%)	1428 (67%)

Supplementary Table 2. Proportions of not being discussed at a multidisciplinary team meeting (MDTM) by age in tertiles and gender.

MeDiC points	All patients OR*(95% CI) (n=8955)	2008-2013 OR*(95% CI) (n=4019)	2014-2019 OR*(95% CI) (n=4936)
11-19	1	1	1
9-10	1.44 (1.24-1.67)	1.47 (1.21-1.79)	1.59 (1.25-2.05)
7-8	1.66 (1.44-1.91)	1.57 (1.30-1.90)	2.24 (1.78-2.83)
3-6	2.11 (1.84-2.43)	1.95 (1.62-2.36)	3.08 (2.46-3.88)
Linear	1.11 (1.09-1.13)	1.09 (1.07-1.12)	1.17 (1.14-1.20)

Supplementary Table 3. Adjusted odds ratios (OR)* (age and gender) for not being discussed at a multidisciplinary team meeting (MDTM) in subgroups by calendar year of diagnosis 2008-2013 and 2014-2019, respectively. ORs are calculated with MeDiC points categorised in quartiles and per one unit decrease of the MeDiC score (linear). CI = Confidence interval; n = numbers
